# Effects of *Bifidobacterium bifidum* in Mice Infected with *Citrobacter rodentium*

**DOI:** 10.3390/microorganisms7020051

**Published:** 2019-02-14

**Authors:** Bijun Wen, Amel Taibi, Christopher R. Villa, Shin-Hann Lee, Sofia Sagaidak, Elena M. Comelli

**Affiliations:** 1 Department of Nutritional Sciences, Faculty of Medicine, University of Toronto, Toronto, ON M5S 1A8, Canada; bijun.wen@mail.utoronto.ca (B.W.); amel.taibi@utoronto.ca (A.T.); christopher.villa@mail.utoronto.ca (C.R.V.); hann.lee@mail.utoronto.ca (S.-H.L.); sofia.sagaidak@mail.utoronto.ca (S.S.); 2 Joannah and Brian Lawson Centre for Child Nutrition, Faculty of Medicine, University of Toronto, Toronto, ON M5S 1A8, Canada

**Keywords:** *Bifidobacterium bifidum* MIMBb75, *Citrobacter rodentium*, inflammatory bowel disease (IBD), EHEC infection, colitis, probiotics

## Abstract

In vitro and in vivo studies suggest that selected *Bifidobacterium bifidum* strains sustain intestinal homeostasis. This study aimed to examine whether the administration of *B. bifidum* MIMBb75 (BB75) attenuates *Citrobacter rodentium* infection, a murine model for enteric infection and inflammatory bowel disease in humans. C57Bl6/J mice were randomized to receive BB75 daily starting before or after *C. rodentium* infection. BB75 load and infection kinetics were monitored. On day 10 post-infection (p.i.), histological parameters of the large intestine were assessed. Barrier integrity was evaluated by pathogen translocation to secondary organs and in vivo permeability test. Fecal *C. rodentium* load peaked at 10^10^ CFU/g at day 10 p.i., with clearance at day 24 p.i., regardless of probiotic treatment. BB75 administration resulted in 10^7^ cells/g of feces with no effect of timing of administration. BB75 treatment did not attenuate *C. rodentium*-induced crypt hyperplasia nor inflammation. *C. rodentium* and BB75 can co-exist in the gut with no mutual displacement. However, BB75 cannot counteract *C. rodentium* pathology. Our findings provide insight for the understanding of probiotics behavior and their clinical relevance in intestinal inflammation.

## 1. Introduction

*Bifidobacterium bifidum* is a Gram-positive anaerobe belonging to the Actinobacteria phylum. *B. bifidum* is one of the first colonizers of the infant gut [1] and members of this species have many health-promoting effects, including immunomodulation [2], bacteriocin production [3], pathogen exclusion [4], and maintenance of health-compatible microbiota [5]. These beneficial effects may have implications in intestinal acute and chronic inflammation, including following enteric infection or in inflammatory bowel disease (IBD). For instance, in vitro, *B. bifidum* LMG13195 sustains the integrity of HT29 cells monolayers [6] and *B. bifidum* ATCC 29521 interferes with attachment and colonization of the foodborne pathogen enterohemorrhagic *Escherichia coli* (EHEC) [7]. In vivo, *B. bifidum* BGN4 alleviates lymphocytes infiltration and Th1-type cytokines production in a naive T-cell transfer mouse model of IBD [8]. *B. bifidum* S17 [9] and, more recently, *B. bifidum* PI22 [10] were shown to dampen inflammation in a trinitrobenzene sulfonic acid-induced colitis mouse model.

*B*. *bifidum* MIMBb75 (BB75) is a human isolate [11] and a probiotic that exhibits strong adhesive properties to intestinal epithelial cells [12], mitigating intestinal discomfort in patients with irritable bowel syndrome [13]. We found that, when administered to mice at 10^8^ colony-forming units (CFU), BB75 is recovered all along the large intestine, with a preference for its proximal regions [14]. BB75 induces intestinal responses at the gene expression level and has immunomodulatory properties [15,16]. In Caco-2 cells, BB75 downregulates expression of *EPAS1* [15], which is a transcription factor activating inflammation during infectious colitis [17]. Another in vitro study reported that the BB75 surface murein lytic enzyme TgaA could induce dendritic cell activation and IL-2 production [16]. In this study, we investigate the ability of BB75 to prevent and/or mitigate intestinal inflammation in mice infected with *Citrobacter rodentium*. *C. rodentium* is a murine enteric pathogen that closely resembles human EHEC pathogenesis and can induce colitis via a Th1/Th17-dominated inflammatory response in the mouse intestine [18]. Thus, *C. rodentium* infection is commonly used as a model for enteric infection and IBD [18]. Probiotic *Lactobacillus* strains and *B. breve* UCC2003 were previously found to mitigate colonic crypt hyperplasia and mucosal inflammation in this model [19,20,21,22,23,24,25]. However, studies focused on the effects of other bifidobacteria species and strains on pathologies associated with *C. rodentium* infection are lacking. It is anticipated that our study will contribute to the growing evidence on the role of probiotics in intestinal inflammation.

## 2. Materials and Methods

### 2.1. Mice and Bacteria

Eighty specific pathogen-free C57Bl6/J male mice, six weeks of age, were obtained from Jackson Laboratories (Bar Harbor, ME, USA) and housed in a containment unit (biosafety level 2), where they received sterile chow and water *ad libitum*. *C. rodentium* DBS100 was generously provided by Dr. Dana Philpott, University of Toronto (originally obtained from Dr. David Schauer). *B. bifidum* MIMBb75 was used as previously described [14]. Animal study design and procedures were approved by the animal ethics committee at the University of Toronto (Animal Use Protocol Number: 20010228) and were in accordance with the Regulations of the Animals for Research Act in Ontario and the Guidelines of the Canadian Council on Animal Care.

### 2.2. Study Design

**Figure 1:**
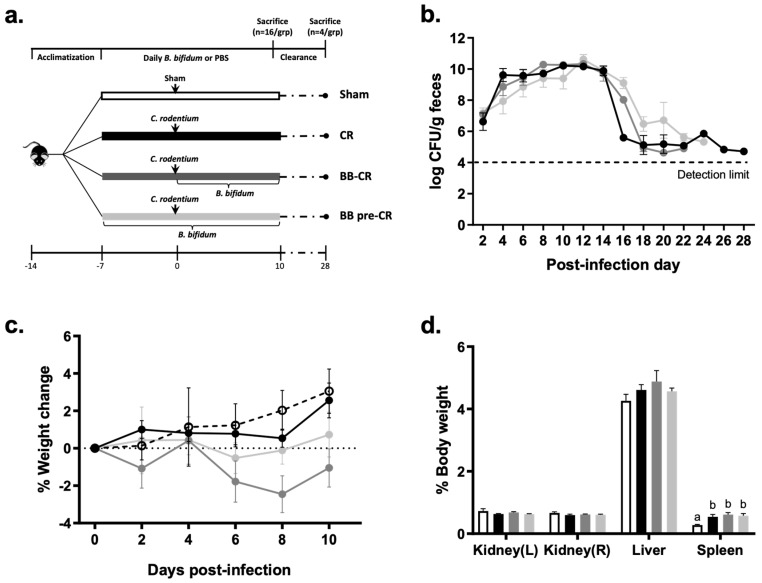
Study design and *C. rodentium* infection model (**a**) study design; (**b**) viable *C. rodentium* counts in infected mice feces were quantified every other day p.i., n = 9–13/group (day 2–10 p.i.), and n = 1–4/group (day 12–28 p.i.); (**c**) percentage of body weight change among groups after infection, n = 12/group; (**d**) percentage of organ weight per gram of body weight among Sham and infected groups on day 10 p.i., n = 8/group. Data are presented as mean ± SE. Statistical significance was determined by repeated-measures ANOVA for *C. rodentium* counts and weight change and one-way ANOVA for organ weight followed by Bonferroni’s post-hoc test. Different superscripts (a,b) indicate significance among groups, *p* < 0.05. (○) Sham, (●) CR, (●) BB-CR, (●) BB pre-CR.

Mice were randomized into four groups (n = 20/group): (1) Sham infected, (2) *C. rodentium* infected (CR), (3) *C. rodentium* infected with daily administration of *B. bifidum* initiated 5 h after, but on the same day as the infection (BB-CR), (4) *C. rodentium* infected with daily *B. bifidum* administration initiated one week (7 days) before the infection (BB pre-CR) (Figure 1a). Infection was performed by intra-gastric gavage of 100 μL LB (lysogeny broth)-cultured *C. rodentium* (10^9^ CFU/mL) or an equal volume of sterile LB (Sham), as previously described [26]. Daily *B. bifidum* treatment was performed by intra-gastric gavage of 200 μL *B. bifidum* suspension in PBS (10^9^ CFU/mL) or an equal volume of sterile PBS.

Body weights were measured and freshly passed fecal pellets were collected on post-infection (p.i.) days 2, 4, 6, 8, 9 and just before sacrifice. A subset of mice (n = 16/group) were sacrificed on day 10 p.i., which is the peak of infection [27], by cervical dislocation after brief exposure to carbon dioxide; caecum (the initial site of infection about 2–3 days p.i.) and distal colon (the major site of infection at day 10 p.i. and defined as the distal 3.5 cm of the colon after excision of the rectum), kidneys, spleen and liver were dissected on ice and fixed in 10% formalin for histology or used for *C. rodentium* quantification as explained below. Four mice per group were retained to monitor the effect of BB75 on *C. rodentium* clearance after day 10 p.i. and thereafter, freshly passed fecal pellets were collected every other day until reaching clearance (below the detection limit for two consecutive days), as previously described [27,28].

### 2.3. Bacteria Culturing and Quantification

For gavage, *C. rodentium* DBS100 was grown in lysogeny broth (Luria-Bertani, LB) aerobically at 37 °C for 16 h, as previously described [26]. Viable counts of *C. rodentium* in liquid culture, gavage suspension, feces, liver and spleen were determined by classical culturing on MacConkey Agar (BioShop); after aerobic incubation for 24 h at 37 °C; *C. rodentium* colonies were identified based on morphology with a detection limit of 10^3^ CFU/g [19].

*B. bifidum* MIMBb75 was grown in an anaerobic chamber (Coy Laboratory Products, Grass Lake, MI, USA) at 37 °C in Man Rogosa Sharpe broth, supplemented with 0.05% L-cysteine hydrochloride (cMRS) for 24 h. The culture was washed and re-suspended in sterile pre-reduced PBS (200 μL) immediately before gavage. Viable counts of *B. bifidum* in gavage culture were enumerated throughout the study with a hemacytometer and by classical culturing on cMRS agar to confirm viability. To assess the fecal load of *B. bifidum*, fecal pellets were collected from uninfected mice 2 days post-*B. bifidum* initiation (i.e., BB pre-CR) and from mice in all groups on day 9 p.i. DNA was extracted using the Omega E.Z.N.A.TM Stool DNA Isolation Kit (Omega Bio-Tek, Norcross, GA), modified as previously described [14]. Quantitative Real-Time PCR was performed with 50 ng of DNA using the SYBR Green Master Mix (Applied Biosystems, Carlsbad, CA) with specific primers targeting the bopA gene, which is specific to the *B. bifidum* species [11] (Forward: 5′ACCGAATTCGCCTGTCACTT3′; Reverse: 5′ACGGCGCGGATTCGT3′). Each reaction was run in triplicates in a 384-well optical plate using a 7900 HT Real-Time PCR machine (Applied Biosystems) with default conditions. Bacterial counts were determined using pre-constructed standard curves, and data were expressed as log10 cells per gram of feces.

### 2.4. Fluorescein Isothiocyanate–Dextran In Vivo Permeability Assay

On day 10 p.i., intestinal permeability was assessed in a subset of overnight fasted mice (n = 8/group) using the fluorescein isothiocyanate (FITC)-dextran (4 kDa, Sigma–Aldrich, Oakville, Canada) assay [26,29]. Serum FITC-dextran concentration was quantified by fluorometry (FusionTM, PerkinElmer) with an excitation wavelength of 485 nm and emission wavelength of 535 nm [26,29].

### 2.5. Histological Analysis

Hematoxylin and Eosin-stained 5 μm tissue sections were used to score inflammation on a scale of 0–4, as previously described [29,30]. A minimum of 1 transverse and 2 longitudinal sections per cecum and distal colon tissue, and 5 far-separated fields per section were assessed to determine the mean mucosal damage score and crypt depths for each mouse with the examiner blinded to the various groups [26,29].

### 2.6. Statistical Analysis

*C. rodentium* fecal counts and body weight changes were analyzed using repeated-measures analysis of variance (ANOVA). Differences in organ weights, crypt hyperplasia, colon damage scores, intestinal permeability, and bacterial translocation were determined by one-way ANOVA followed by the Bonferroni’s post-hoc test as necessary. The difference of fecal *B. bifidum* counts before and after infection and between *B. bifidum*-treated groups were assessed by Student’s t-test. A P-value of P < 0.05 was considered statistically significant. All statistical analyses were performed with Prism 7.0d software (GraphPad Software, La Jolla, CA, USA).

## 3. Results

### 3.1. C. rodentium Infection Kinetics and Characteristics

*C. rodentium* was detectable in the feces two days after infection in all infected groups; viable counts increased until reaching a plateau (10^10^ CFU/g) at day 10 p.i. (Figure 1b), then decreased rapidly and reached clearance on approximately day 24 p.i. on average, with no significant differences among groups. The infection did not result in significant changes of body weight among groups (Figure 1c). Spleen weights were significantly higher in the infected groups compared to the Sham but did not differ among treatments (CR: 0.54 ± 0.075, BB-CR: 0.61 ± 0.06, BB pre-CR: 0.58 ± 0.07, Sham: 0.28 ± 0.02% body weight; p < 0.01); there was no significant difference in kidney and liver weights among groups (Figure 1d).

### 3.2. B. bifidum Recovery in Feces

**Figure 2:**
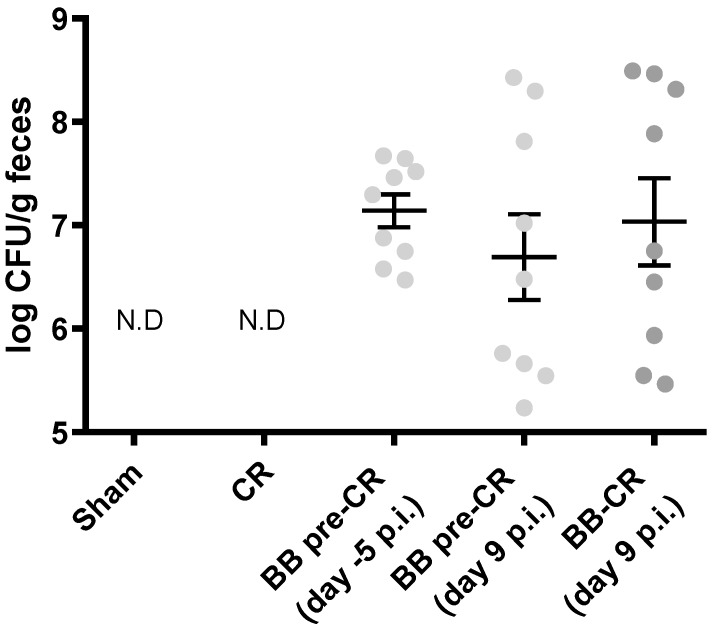
Fecal BB75 load before and after infection. Real-time PCR quantification of fecal BB75 in all groups of mice (n = 9/group) after *C. rodentium* (CR) infection, and in BB pre-CR group two days after initiation of BB75 (i.e., five days before infection). Statistical significance was determined by Student’s t-test, data presented as mean ± SE. N.D, not detectable.

Before infection, *B. bifidum* load in feces of the BB pre-CR mice after day 2 of administration was 7.2 ± 0.13 log cell/g and there was no significant difference in *B. bifidum* load before (−5 p.i.) and after infection (9 p.i.) in this group. On day 9 p.i, there was also no significant difference in *B. bifidum* load between the two probiotic-treated groups (BB-CR: 7.0 ± 0.4, BB pre-CR: 6.7 ± 0.4 log cell/g) (Figure 2). *B. bifidum* was not detected in CR and Sham mice, which did not receive *B. bifidum* treatment.

### 3.3. Intestinal Morphology and Histopathology

**Figure 3:**
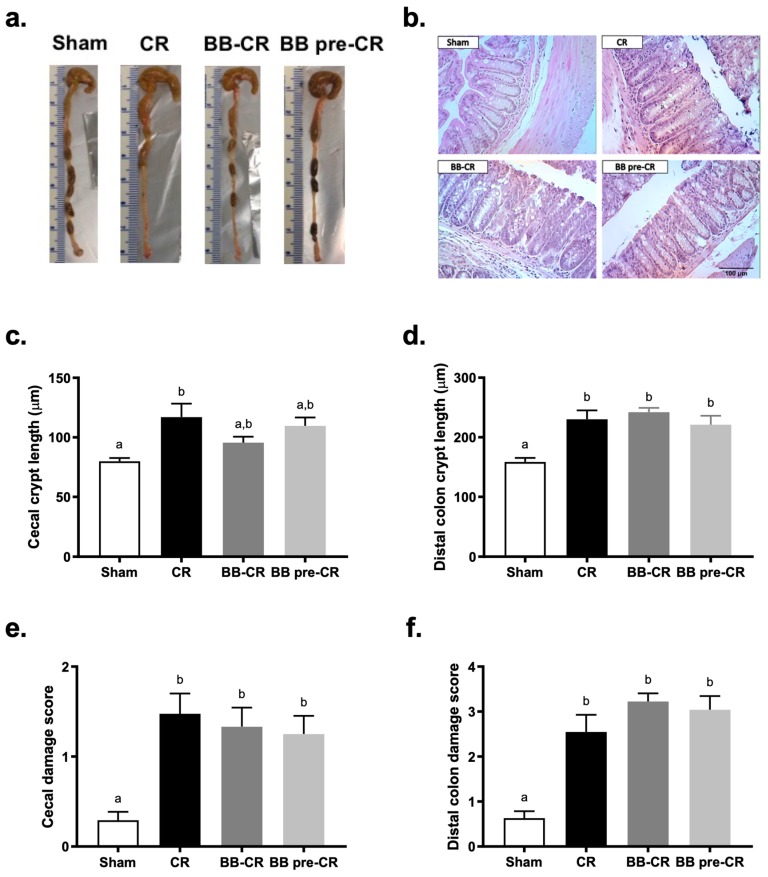
The effect of BB75 on intestinal crypt hyperplasia and tissue damage at day 10 p.i. (**a**) colon morphology; (**b**) representative H&E stained histology of distal colon tissues on day 10 p.i.; (**c**) ceacal (n = 7–8/group) and (**d**) distal colon crypt lengths (n = 10/group); (**e**) ceacal and (**f**) distal colon damage score (n = 10/group) were accessed on a scale of 0–4 (0, no signs of inflammation; 1, minimal evidence of inflammatory cell infiltration; 2, significant evidence of inflammatory cell infiltration; 3, significant evidence of inflammation with goblet cell depletion; 4, sever inflammation characterized by widespread inflammatory cells infiltration, goblet cell depletion and destruction of the mucosal architecture). Results are expressed as mean ± SE. Statistical significance was determined by one-way ANOVA, followed by the Bonferroni’s post-hoc test. Different superscripts (a,b) indicate statistical significance between groups, *p* < 0.05.

Macroscopic observation of the large intestine morphology revealed that infected mice had enlarged cecum and loose intestinal content compared to the Sham; BB75 treated mice did not exhibit apparent differences compared to the untreated mice (Figure 3a). Histopathology analysis showed that *C. rodentium* infected mice exhibited a significant increase of crypt length in the cecum and distal colon, compared to the Sham (Figure 3b–d). Cecal crypt length of mice receiving BB75 did not differ significantly from Sham nor infected controls, suggesting a marginal but not significant normalization effect of BB75 in the cecum. However, distal colon crypt lengths of mice receiving BB75 were significantly greater than Sham control, but did not differ from infected controls. Histological analysis of the cecum and distal colon tissue revealed significant difference in tissue damage scores between infected mice and Sham mice, but no effect of *B. bifidum* administration (Figure 3b,e,f).

### 3.4. Barrier Integrity

**Figure 4:**
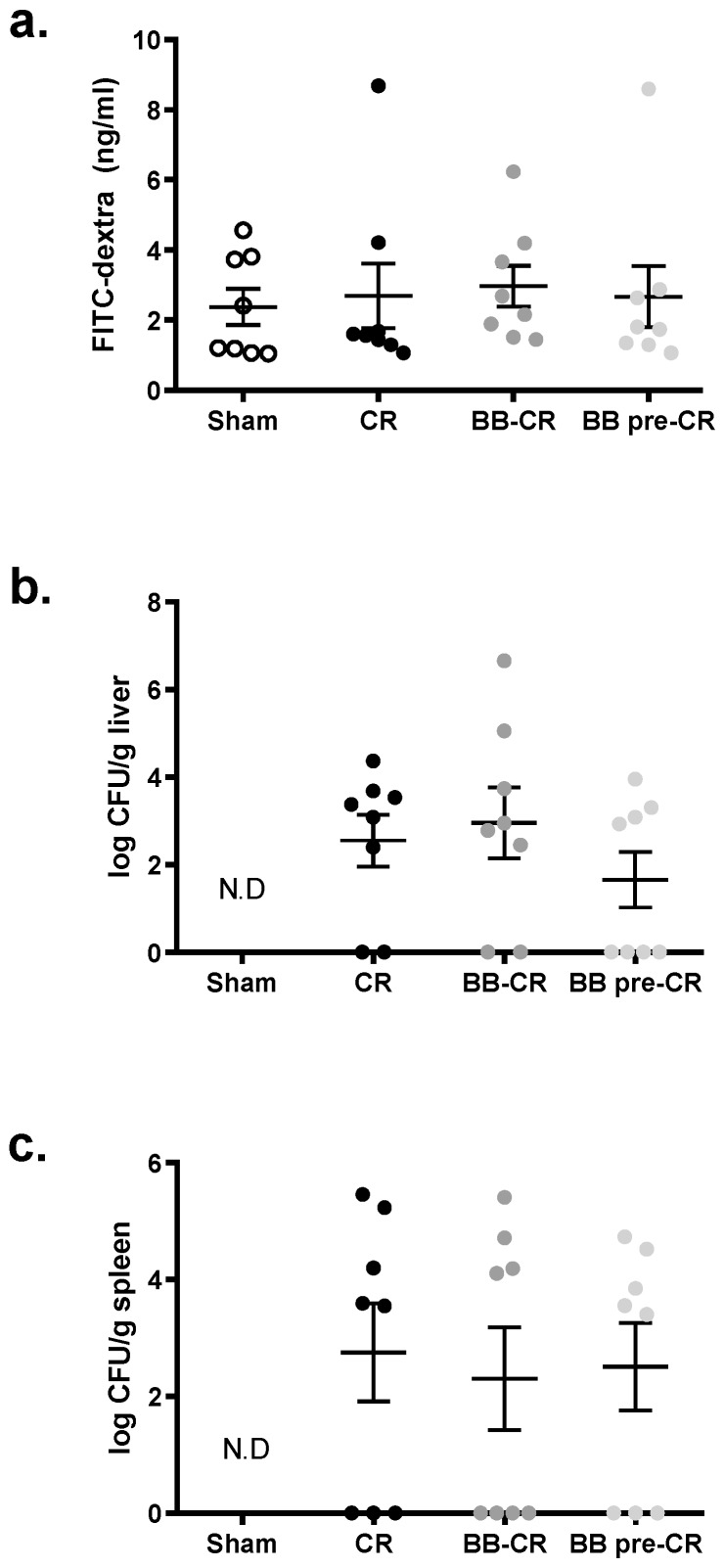
The effect of BB75 on the intestinal barrier at day 10 p.i. (**a**) In vivo FITC-dextran intestinal permeability test (n = 8/group); no difference was detected in intestinal permeability among the groups, despite *C. rodentium* or BB75 treatment; *C. rodentium* translocation to (**b**) liver and (**c**) spleen (n = 8/group); no difference in *C. rodentium* translocation was detected among groups. Results are presented as mean ± SE. Statistical significance was determined by one-way ANOVA. N.D, not detectable.

There was no difference in intestinal permeability measured by the FITC-dextran assay among the four groups on day 10 p.i. (Figure 4a). *C. rodentium* was quantifiable in the liver and spleen of infected but not control mice; though, *B. bifidum* treatment did not affect these counts (Figure 4b,c).

## 4. Discussion

This study investigated the impact of probiotic *B. bifidum* MIMBb75 on *C. rodentium* infection. The probiotic could be recovered in the feces of the infected mice. Though, its administration in either a preventative or treatment manner did not improve intestinal pathology (*C. rodentium* colonization, crypt hyperplasia, inflammation, and barrier dysfunction).

BB75 was quantified in the feces at day 9 p.i. and counts were found to align with the fecal load in healthy mice [14]; moreover, fecal BB75 counts in BB pre-CR group before and after infection (Figure 2) were not significantly different. This indicates that infection did not interfere with *B. bifidum* fecal recovery. *C. rodentium* is only pathogenic in murine and can replace almost 90% of the resident microbiota [18,31]. The fact that BB75, a human-restricted strain, was not displaced, is in line with reports showing the excellent gut colonization capacity of this strain [11].

Selected probiotic bacteria were found to result in a reduction of *C. rodentium* load during infection [19,20,21,22,23,24,25]. For example, treatment of *L. rhamnosus* R0011 and *L. acidophilus* R0052 reduced *C*. *rodentium* attachment to T84 epithelial cells in vitro, and its load in colonic luminal contents at day nine p.i. in C57Bl/6 mice [19]. A three-day pre-treatment of *B. breve* UCC2003 in BALB/c mice reduced fecal *C. rodentium* load from day 3 to 14 p.i. [24]. In the present study, BB75 administration did not suppress *C. rodentium* colonization nor did it improve its clearance, suggesting that the two strains can co-exist in the gut ecosystem with no mutual exclusion. Since we previously showed that BB75 predominately localizes to the cecum rather than to the distal colon [14], where *C. rodentium* preferentially resides, future studies may examine colonization competition between the two bacteria in a region-specific manner.

Competition for colonization sites within the intestine is not always necessary for probiotics to mitigate *C. rodentium* pathology. For example, treatment with *B. breve* UCC2003 for three days prior to infection attenuated crypt hyperplasia without affecting *C. rodentium* colonization or A/E lesion formation in C57Bl/6 mice [25]. A more recent study conducted by this group also found that administration of fermented product from *Lactobacillus* species to C57Bl/6 mice improved infection, but did not alter *C. rodentium* colonization kinetics [22]. Yet, in the present study, TMCH and histological damage of the cecum and distal colon were not attenuated in mice treated with BB75 whether before or after *C. rodentium* challenge. To our knowledge, no studies are available of *B. bifidum* in a *C. rodentium*-induced colitis model. It is known that the gene expression program of BB75 changes in response to different local environments. For example, the adhesion efficiency of BB75 was found to vary across various differentiation stages of epithelial cells [32]. It was also shown that the expression level of pili (adhesion factors) in other *B. bifidum* strains varies according to the available carbon source [33]. Thus, the ability of BB75 to attenuate infection may depend on the environmental conditions, and may differ in the human ecosystem. It is also possible that this species, or this particular strain, is not the optimal choice in this context. Finally, it is possible that the conditions chosen for this study mask benefits. We used a very well established and reproducible *C. rodentium* infection protocol [26,29], which has been employed in the past for probiotic studies [19,20,21,22,23]. However, we cannot exclude that BB75 may have beneficial effects in other colitis models. While we used live probiotic cells, which is in accordance with the current definition of probiotic [34], it is also possible that BB75 administered as a post or para-probiotic, may elicit different effects.

One unexpected observation in the present study was the general increase of intestinal permeability in all mice including those in the Sham-infected group with no differences among groups. It is known that *C. rodentium* infection results in intestinal barrier dysfunction and therefore, increased paracellular translocation of the macromolecular tracer FITC-dextran [21,29]. For example, Rodrigues et al. showed that on day 10 p.i. serum FITC-dextran concentration in infected adult mice was two-times higher than that of the Sham mice [21], which we have also observed in a recent study [29]. Surprisingly, serum FITC-dextran concentration of the Sham mice in the present study was as high as the infected groups on day 10 p.i, suggesting impaired barrier integrity in healthy uninfected mice. Mice in our study underwent daily intra-gastric gavage for 17 days. This technique allows to control for the amount of probiotic received by each mouse, but it imposes physical and psychological stress to the animals. Chronic psychological stress can trigger the release of the glucocorticoid hormone and lead to mucosal barrier dysfunction, thereby increased intestinal permeability and host defense mechanism impairment [35]. In fact, studies that showed positive effects of probiotic treatment with normal ranges of intestinal permeability in the Sham controls, administered probiotics daily in drinking water [19,20,21]. Other studies used prolonged daily gavage of probiotics, but did not measure permeability [25]. In line with this, *C. rodentium* translocation to secondary organs, spleen, and liver, was similar among the infected groups, indicating similar alteration of the barrier integrity.

## 5. Conclusions

In summary, this is the first study addressing the effects of BB75 in infectious colitis. It was found that administration of this strain did not attenuate *C. rodentium* colonization, intestinal inflammation, crypt hyperplasia and barrier dysfunction. Findings from this study may provide insights for future investigation, particularly with respect to the protocol in which probiotics are administered. From a translational perspective, this study may help in the selection of probiotics to be tested in the clinical setting [36,37], at least in the context of Crohn’s disease, and provides further evidence that probiotics benefits cannot be extrapolated across different conditions.
